# The new European Society of Cardiology guideline for the management of cardiomyopathies: key messages for cardiac electrophysiologists

**DOI:** 10.1007/s00399-023-00975-y

**Published:** 2023-11-16

**Authors:** Dennis Korthals, Lars Eckardt

**Affiliations:** Department of Cardiology II: Electrophysiology, University Hospital Münster, Albert-Schweitzer-Campus 1, 48149 Münster, Germany

**Keywords:** Cardiomyopathy, Guidelines, Arrhythmia, Electrophysiology, Sudden cardiac death, Risk stratification, Kardiomyopathie, Leitlinien, Arrhythmie, Elektrophysiologie, Plötzlicher Herztod, Risikostratifizierung

## Abstract

Electrocardiographic findings and arrhythmias are common in cardiomyopathies. Both may be an early indication of a specific diagnosis or may occur due to myocardial fibrosis and/or reduced contractility. Brady- and tachyarrhythmias significantly contribute to increased morbidity and mortality in patients with cardiomyopathies. Antiarrhythmic therapy including risk stratification is often challenging and plays a major role for these patients. Thus, an “electrophysiological” perspective on guidelines on cardiomyopathies may be warranted. As the European Society of Cardiology (ESC) has recently published a new guideline for the management of cardiomyopathies, this overview aims to present key messages of these guidelines. Innovations include a new phenotype-based classification system with emphasis on a multimodal imaging approach for diagnosis and risk stratification. The guideline includes detailed chapters on dilated and hypertrophic cardiomyopathy and their phenocopies, arrhythmogenic right ventricular cardiomyopathy, and restrictive cardiomyopathy as well as syndromic and metabolic cardiomyopathies. Patient pathways guide clinicians from the initial presentation to diagnosis. The role of cardiovascular magnetic resonance imaging and genetic testing during diagnostic work-up is stressed. Concepts of rhythm and rate control for atrial fibrillation have led to new recommendations, and the role of defibrillator therapy in primary prevention is discussed in detail. Whilst providing general guidelines for management, the primary objective of the guideline is to ascertain the disease etiology and disease-specific, individualized management.

## Abstract (de)

Arrhythmien und EKG(Elektrokardiographie)-Auffälligkeiten sind bei Kardiomyopathien häufig. Sie können ein erster Hinweis auf eine spezifische Diagnose sein oder im Verlauf einer Erkrankung als Folge von Fibrosierung und/oder reduzierter Herzfunktion auftreten. Brady- und Tachyarrhythmien tragen dabei signifikant zu einer erhöhten Morbidität und Mortalität bei Patienten mit Kardiomyopathien bei. Eine antiarrhythmische Therapie einschließlich einer Risikostratifizierung ist oft eine Herausforderung und bei der Behandlung dieser Patienten von besonderer Bedeutung. Daher ist eine *elektrophysiologische* Sicht auf Empfehlungen zu Kardiomyopathien sinnvoll. Da die ESC (European Society of Cardiology) kürzlich eine neue Leitlinie zum Management von Kardiomyopathien veröffentlichte, werden in der vorliegenden Arbeit deren wichtigsten Empfehlungen mit besonderem Fokus auf der kardialen Elektrophysiologie vorgestellt. Innovationen umfassen eine neue phänotypbasierte Klassifikation mit Schwerpunkt auf multimodaler Bildgebung zur Diagnostik und Risikostratifizierung. Die aktuelle Leitlinie enthält ausführliche Kapitel zur dilatativen und hypertrophen Kardiomyopathie und deren Phänokopien, der arrhythmogenen rechtsventrikulären Kardiomyopathie, der restriktiven Kardiomyopathie sowie syndromalen und metabolischen Kardiomyopathien. Pfade leiten von der Erstvorstellung bis zur Diagnose und Therapie. Die Rolle der kardiovaskulären Magnetresonanztomographie und der genetischen Diagnostik erfährt einen besonderen Stellenwert. Konzepte zur Rhythmus- und Frequenzkontrolle bei Vorhofflimmern führten zu neuen Empfehlungen, und die Rolle der Defibrillatortherapie in der Primärprävention wird ausführlich diskutiert. Das Hauptziel der neuen Leitlinie ist, die Krankheitsursachen bestmöglich zu ermitteln, um eine spezifische, individualisierte Behandlung zu ermöglichen.

Electrocardiographic findings and arrhythmias are closely related to cardiomyopathies (CM). Both may be an early indication of a specific diagnosis or are (just) the consequence of structural heart disease with myocardial fibrosis and impaired contractility. Arrhythmias significantly contribute to increased morbidity and mortality, and CM are the leading cause of exercise induced syncope and sudden cardiac death (SCD) in young people (Fig. 1). Antiarrhythmic therapy including risk stratification in CM is often challenging and plays a major role in cardiac electrophysiology. Thus, an “electrophysiological” perspective on CM guidelines may be warranted. As the European Society of Cardiology (ESC) has recently published a new guideline for the management of CM [1] this overview aims to present key aspects of this guideline.

**Fig. 1:**
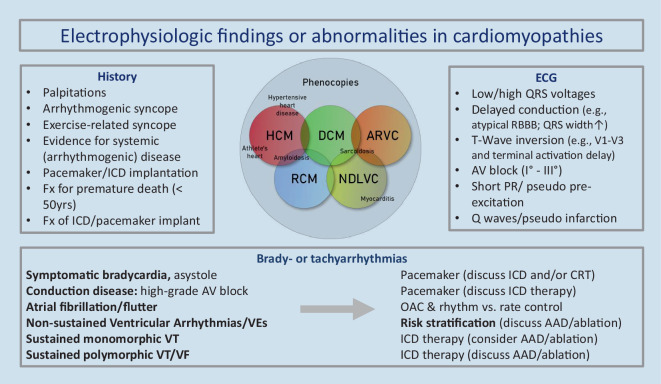
Characteristics of cardiomyopathies focusing on history of arrhythmias, electrocardiographic and electrophysiological findings with selected therapeutic options. *Fx* family history, *ICD* implantable cardioverter defibrillator, *RBBB* right bundle branch block, *CRT* cardiac resynchronization therapy, *OAC* oral anticoagulation, *VEs* ventricular extrasystoles, *AAD* antiarrhythmic drugs, *VT/VF* ventricular tachycardia/fibrillation

## Brief overview of the new guidelines on cardiomyopathies

Cardiomyopathies (CM) are common and may lead to heart failure and life-threatening ventricular arrhythmias (VA). Prevalence and incidence of arrhythmias are difficult to assess, as disease expression varies throughout life. Geographical distribution of genetic variants also influences the prevalence in different populations, ethnicities, regions, and countries. Hypertrophic cardiomyopathy (HCM) for example has got an estimated overall prevalence of 0.2%, while for dilated cardiomyopathy (DCM) estimates are wider ranging from 0.036% to 0.4% [1]. In the past, CM were mainly classified by anatomical and clinical characteristics, using echo- and electrocardiography, invasive angiography, and histology. Nowadays, knowledge about CM has expanded exponentially by utilizing new imaging approaches and molecular technologies. For the first time, the new ESC guideline addresses CM other than HCM. It includes detailed chapters on HCM, DCM, arrhythmogenic right ventricular cardiomyopathy (ARVC), restrictive cardiomyopathy (RCM) including cardiac amyloidosis (CA) as well as syndromic and metabolic CM including Anderson-Fabry disease but also discusses important differential diagnoses such as cardiac sarcoidosis. The new term “non-dilated left ventricular cardiomyopathy” (NDLVC) replaces “hypokinetic non-dilated cardiomyopathy.” It is defined “as the presence of non-ischemic LV scarring or fatty replacement regardless of the presence of global or regional wall motion abnormalities, or isolated global LV hypokinesia without scarring.” Thus, the diagnosis is predominantly based on cardiac magnetic resonance imaging (CMR).

## General aspects of arrhythmias in cardiomyopathies

**Fig. 2:**
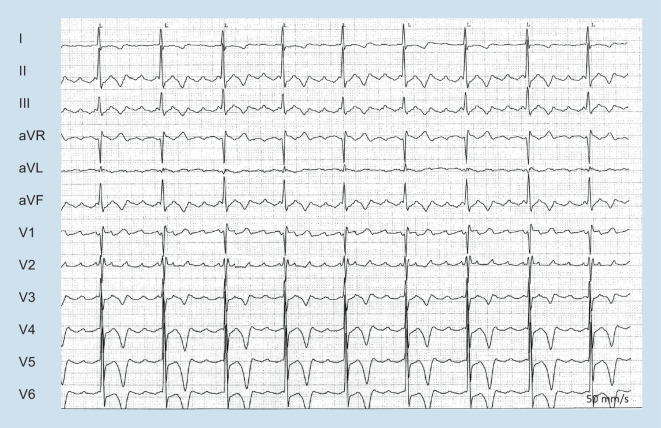
Typical atrial flutter in a 69-year-old male with apical hypertrophic cardiomyopathy (septal thickness of 16 mm). Please note the electrocardiographic signs of left ventricular hypertrophy with high R waves going along with prominent negative T‑waves in V4–V6

The spectrum of arrhythmias ranges from bradyarrhythmia, atrial and/or ventricular extrasystoles (VEs) to atrial fibrillation/flutter (Fig. 2) and/or life-threatening VA [2]. Atrial fibrillation (AF) represents the most common arrhythmia and is associated with an increased risk of cardioembolic events, heart failure, and death in patients with CM. In parallel to AF patients without CM, there is increasing evidence for AF ablation in an era of a steady increase in catheter ablations [3, 4]. The prevention of SCD plays an important role in the new recommendations. The guideline includes five class I indications for ICD therapy in CM (all secondary prevention) and is in line with the 2022 ESC Guidelines for the management of patients with VA and the prevention of SCD [2, 5, 6]. It expands the previously established concept of individualized decision-making and supports etiology-specific individual management and risk stratification. Recommendations for genetic testing have increased (seven class I recommendations!) and the guideline acknowledges the growing evidence on certain high-risk genotypes regardless of LV morphology and function. This includes, for example, Class I recommendations for genetic testing in all patients fulfilling diagnostic criteria for CM as well as genetic cascade testing of relatives in case of a pathological or likely pathological variant in the index patient (Class I). Next to genetic testing, the role of CMR has increased in recent years [7] but still lacks randomized trials to guide ICD therapy. Thus, the ESC guideline stresses the importance of a tailored, etiology-based individual management approach incorporating the patient’s history, electro- and echocardiography, CMR, laboratory analysis, genetics, as well as electrophysiological findings/studies.

## A standardized pathway after initial presentation and the role of electrocardiography in cardiomyopathies

The proposed systematic diagnostic approach includes characterization of a phenotype with multimodality imaging. In addition to routine echo- and electrocardiography [8], CMR characterizes myocardial tissue and identifies edema and/or scarring [9–14]. The distribution and severity of interstitial expansion, as visualized by late gadolinium enhancement (LGE), may suggest a specific diagnosis [7]. As LGE patterns have been shown to be prognostic for VA in various CM, the guideline also acknowledges the role of CMR in risk stratification [15, 16]. A key message is the Class I recommendation for all individuals with a CM to undergo contrast-enhanced CMR during the initial assessment and during follow-up to detect disease progression. Of note, the interpretation of imaging results should always consider the clinical context, including personal and family history, clinical presentation, for example, arrhythmias and other more specific tests such as genetic testing if an inheritable CM is suspected.

**Fig. 3:**
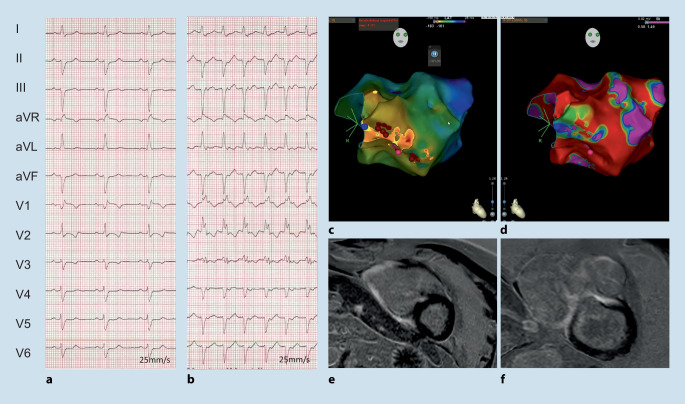
A 49-year-old male with biopsy-proven cardiac sarcoidosis. **a** Atypical right bundle branch block (RBBB) with prominent second component of the QRS complex in the presence of atrioventricular block I. **b** Sustained hemodynamically tolerated ventricular tachycardia (CL 430 ms). **c**–**f** Electro-anatomical CARTO activation map and bipolar right ventricular voltage map with extensive fibrosis that correlated with the cardiac magnetic resonance imaging illustrated by late gadolinium enhancement

A resting 12-lead electrocardiogram (ECG) is often an early diagnostic step. ECG abnormalities in patients with CM are common and can occur years before overt morphological or functional abnormalities (Fig. 1). Most ECG abnormalities are nonspecific. For example, epsilon waves may occur in ARVC but also in other CM such as cardiac sarcoidosis (Fig. 3; [17, 18]). Recently, an easily applicable algorithm including PR prolongation and the surface area of the maximum R’ wave in leads V1 through V3 has been demonstrated to distinguish cardiac sarcoidosis from ARVC. This QRS terminal activation in precordial leads V1 through V3 may reflect disease-specific scar patterns [19]. Unspecific ECG abnormalities should always be interpreted in conjunction with imaging findings, patient history, and clinical features and may provide important clues to the underlying diagnosis [17]. Some features, such as atrioventricular (AV) block, pseudo pre-excitation, low QRS voltages, or the distribution of repolarization changes may also be helpful (Fig. 1). Accordingly, the guideline recommends acquiring an ECG at the first visit from all patients with suspected or known CM and during regular follow-up. As arrhythmias are common in CM, ambulatory ECG monitoring at initial presentation and in regular intervals (e.g. every 1–2 years in stable patients [Class I]) is also advised for stroke and SCD risk stratification. On an individual basis a biopsy may be helpful [20, 21], but is only specifically mentioned for RCM (Class IIa).

## Atrial fibrillation in cardiomyopathies

AF is by far the most common sustained arrhythmia in patients with CM and associated with a worse prognosis, i.e., increased mortality and incidence of stroke [22]. Early-onset AF may be an early marker for an underlying syndromic or metabolic cardiomyopathy. A CM may on the other hand be an important predisposing factor for AF in the elderly [23]. The EURObservational Research Programme (EORP) CM registry [24, 25] demonstrated an AF prevalence of 28% at baseline (with an overall annual incidence of 3%) with more severe symptoms and worse prognosis for CM patients with AF. The incidence of stroke was increased threefold as compared to the general population. Short atrial runs may better predict AF in CM patients than in other patients but this needs to be evaluated, for example, with loop recorders [26]. Although the “Atrial Fibrillation Better Care (ABC)” approach [25, 27] has not been comprehensively assessed in patients with CM, two randomized trials (the RACE-III trial [28] and the mobile Atrial Fibrillation App Trial (mAFA-II trial) [29, 30]) provided some evidence in favor of an integrated approach in CM. Thus, the guideline recommends modification of risk factors such as obesity, reduced physical activity, as well as alcohol consumption, hypertension, diabetes, and chronic obstructive pulmonary disease in CM patients with recurrent AF (Class I). CM seem to have different thromboembolic risks. Several observational studies reported an increased risk in HCM, RCM, and cardiac amyloidosis. In an unselected transesophageal echocardiography population, 20% of AF patients with a left atrial appendage (LAA) thrombus had a CHA_2_DS_2_-VASc score < 2 [31] and there is evidence suggesting that the CHA_2_DS_2_-VASc score, which has not been validated for CM patients, may insufficiently assess thromboembolic risks in patients with HCM, RCM, or cardiac amyloidosis. Thus, the current guideline recommends oral anticoagulation (OAC) for all patients with HCM and cardiac amyloidosis and AF (Class I) and gives a Class IIa recommendation for RCM. In contrast, for AF patients with DCM, NDLVC, or ARVC, anticoagulation is recommended utilizing the CHA_2_DS_2_-VASc score for risk stratification (Class I). As in patients without CM, non-vitamin K-dependent OAC (NOAC) is preferred, except in patients with severe mitral stenosis and/or mechanical valve replacement.

Acknowledging the lack of consistent data for CM patients, rate control may be considered for all patients with AF, especially as AF-induced cardiac dysfunction may worsen the underlying CM [32]. As diastolic filling is reduced with increasing heart rates, AF can result in hemodynamic and clinical decompensation. This can be especially relevant for CM patients. In parallel to AF patients without CM, a lenient rate control (resting < 110 bpm) may be sufficient, but observational studies [33, 34] also suggest that CM patients with heart failure may benefit from lower heart rates. As there is no reliable data regarding the choice of pharmacological rate control in CM patients the guideline recommends beta-blockers as the preferred agent due to their established safety profile. Digoxin may be considered as an alternative; however, caution is advised due to potentially harmful effects in particular in combination with antiarrhythmic drugs (AAD) [35, 36] and inconsistent data regarding mortality in observational studies [37, 38]. In patients with left ventricular outflow tract obstruction (LVOTO), digoxin should be avoided (Class IIa). Non-dihydropyridine calcium channel blockers such as verapamil or diltiazem should only be used in CM patients with LVEF ≥ 40% though verapamil may be beneficial in combination with Class III AAD in reducing proarrhythmia [39]. AAD therapy is mostly limited to amiodarone and sotalol [40] in CM patients with significantly reduced left ventricular function (HFrEF) or significant left ventricular hypertrophy. For some CM such as ARVC [41], there is evidence that patients may benefit from flecainide [42]. In the presence of an implantable cardioverter (ICD), an individualized AAD strategy balancing risks and benefits of, for example, class III vs. class I AAD should be considered. In the case of insufficient rate control, AV-node ablation with concomitant pacemaker or preferably CRT is an alternative for selected patients (e.g., unsuitable for ablation or failed ablation) [43] and may promise symptomatic improvement in those aged > 75 years [44]. Perspectively, the potential benefit of conduction system pacing compared to CRT after AV-node ablation has to be identified [45].

## Benefits of rhythm versus rate control in cardiomyopathies

More recently, the benefits of rhythm control over rate control have been extensively discussed for symptomatic and asymptomatic [46] patients with AF [47, 48]. In the EAST trial, attaining sinus rhythm seemed to be the key mediator for reduction in cardiovascular outcomes in patients with recently diagnosed AF and cardiovascular risk factors [49]. Patients with AF at the 12-month follow-up visit did not benefit from rhythm control during the remaining 4 years of follow-up. Whether these results are transferable to CM has not been investigated yet. Nevertheless, the current guideline prefers rhythm control with maintaining sinus rhythm at an early stage, especially in symptomatic CM patients with limited success of long-term AAD therapy and known relevant clinical and experimentally proven side effects [50–52], but also for asymptomatic patients without major risk factors for AF recurrence (Class IIa). AF ablation should be considered as first-line therapy to improve symptoms in selected CM patients (Class IIa). For patients with genetic CM (other than HCM) the guideline recognizes a lack of data whilst at the same time emphasizing the potential of proarrhythmia when using class I antiarrhythmic drugs [53, 54] in the presence of structural abnormalities in CM patients. Consequently, catheter ablation is recommended as a safe and superior alternative to AAD for rhythm control, alleviating symptoms, and improving quality of life, and in selected patients to improve survival and reduce heart failure hospitalizations (Class IIa). In addition, AF ablation is recommended for all symptomatic CM patients after failed AAD therapy or in case of tachycardia-induced LV dysfunction regardless of symptoms (Class I). When comparing AF ablation to AAD in patients with heart failure, several trials have demonstrated impressive benefits in favor of ablation [55]. For patients suffering from heart failure with preserved ejection fraction (HFpEF) some observational ablation studies have also suggested a benefit in AF burden and mortality [56].

The role of AF ablation in CM subtypes has been investigated in several registries, mostly in HCM patients and to a lesser extent for rarer diseases such as cardiac amyloidosis [57–59] or sarcoidosis [60]. In HCM (see below), rhythm control is achieved in about two thirds of patients; however, repeat procedures and/or continuation of AAD is often needed. CM patients have a higher risk of AF recurrences, particularly in the presence of atrial fibrosis/remodeling, for example, in patients with HCM, a laminopathy, or cardiac amyloidosis. In HCM patients with planned septal myectomy, surgical ablation and/or LAA occlusion/resection may be considered (Class IIb). Further studies will have to clarify who benefits most, which technology should be used (single tip, vs. balloon vs. variable loop catheters [3, 61]), and whether, for example, gender differences [62] regarding outcome of antiarrhythmic therapy are present in CM.

## Management of ventricular arrhythmias and prevention of sudden cardiac death in cardiomyopathies

Data regarding management of VA in patients with specific genetic cardiomyopathies is scarce; however, the new ESC guideline illustrates some general concepts. Referencing the 2022 ESC Guidelines for the management of patients with VA and the prevention of SCD [2, 5, 6], the Task Force highlights the importance of identifying reversible and precipitating factors. Acute termination of sustained VA should be accomplished by cardioversion, AAD, or pacing, depending on hemodynamic tolerance, etiology, and patient profile. In a patient with electrical storm, mild-to-moderate or even deep sedation should be considered. If patients are not responding to AAD therapy, catheter ablation is recommended. In refractory cases, autonomic modulation/mechanical circulatory support can be considered. For prevention of VA recurrences, AAD (in scar-related VA mostly limited to beta blockers, amiodarone, or sotalol [40]), and catheter ablation (especially for sustained monomorphic VA, or polymorphic VA triggered by VEs of similar morphology) are indicated.

Shared-decision making that is evidence-based and takes individual preferences, circumstances, and values into account, guides ICD recommendations (Class I). An ICD is only reasonable when good quality survival of longer than 1 year is expected. Furthermore, counseling about inappropriate shocks, implant complications, and social, occupational, and driving implications is obligatory before implantation (Class I). For secondary prevention, ICD implantation is recommended irrespective of CM phenotype (Class I). For primary prevention (Table 1), a comprehensive SCD risk stratification is recommended at initial presentation and at 1‑ to 2‑year intervals, or whenever there is a change in clinical status (Class I). The use of validated risk scores (Table 2) to aid decision-making is recommended in HCM (Class I) and should be considered in DCM, NDLVC, and ARVC (Class IIa). It must always be evaluated whether a patient might profit from CRT (Class I). Given the expected need for CMR during follow-up, simpler ICD devices (e.g. single chamber or subcutaneous [63–65] coils in particular in children [66]) should be considered. Data on benefits of the wearable cardioverter defibrillator for primary prevention, for example, in myocarditis or peripartum CM, are so scarce that no recommendations are given.

**Table 1:** Class I and Class IIa recommendations for implantable cardioverter defibrillator therapy in primary prevention of sudden cardiac death (SCD) in patients with cardiomyopathy. Modified from [1]

Recommendations	Class
**General recommendations**	
*The use of validated SCD algorithms/scores as aids to the shared decision-making when offering ICD implantation, where available*	
Is recommended in patients aged ≥ 16 with HCM (see Table 2)	I
Should be considered in patients with DCM, NDLVC, and ARVC	IIa
*If a patient with cardiomyopathy requires pacemaker implantation, comprehensive SCD risk stratification to evaluate the need for ICD implantation should be considered*	IIa
**Choice of ICD**	
*When an ICD is indicated, it is recommended to evaluate whether the patient could benefit from CRT*	I
*Subcutaneous defibrillators should be considered as an alternative to transvenous defibrillators in patients with an indication for an ICD when pacing therapy* *for bradycardia, cardiac resynchronization, or antitachycardia pacing is not anticipated*	IIa
**Hypertrophic cardiomyopathy (HVM)**	
*It is recommended that the 5‑year risk of SCD be assessed at first evaluation and re-evaluated at 1‑ to 2‑year intervals or whenever there is a change in clinical status*	IIa
*Implantation of an ICD should be considered in patients with an estimated 5‑year risk of sudden death of ≥* *6%, following detailed clinical assessment considering:* (i) The lifelong risk of complications (ii) Competing mortality risk from the disease and comorbidities, AND (iii) The impact of ICD on lifestyle/socio-economic status/psychological health	IIa
*In patients with LV apical aneurysms, decisions about primary prevention ICD based on an assessment of risk using the HCM Risk-SCD or a validated pediatric risk-prediction (e.g., HCM Risk-Kids) tool and not solely on the presence of the aneurysm should be considered*	IIa
**Dilated and non-dilated left ventricular cardiomyopathy**	
*An ICD should be considered to reduce the risk of SCD and all-cause mortality in patients with DCM/NDLVC, symptomatic heart failure, and LVEF ≤* *35% despite >* *3 months of OMT*	IIa
*The patient’s genotype should be considered in the estimation of SCD risk in DCM/NDLVC (see *Table 2*)*	IIa
*An ICD should be considered in patients with DCM/NDLVC with a genotype associated with high SCD risk and LVEF >* *35% in the presence of additional risk factors*.^b^	IIa
**Arrythmogenic right ventricular cardiomyopathy (ARVC)**	
*High-risk features*^*c*^* should be considered to aid individualized decision-making for ICD implantation in patients with ARVC*	IIa
*The updated 2019 ARVC risk calculator should be considered to aid individualized decision-making for ICD implantation (see *Table 2*)*	IIa

^a^Shared decision-making is greatly enhanced by patient decision aids tailored specifically to receivers of care as well as more traditional decision-support tools for healthcare practitioners

^b^Additional risk factors in DCM/NDLVC include syncope, LGE presence on CMR

^c^High-risk features in ARVC: arrhythmic syncope, NSVT, RVEF < 40%, LVEF < 45%, SMVT at PES

**Table 2:** Validated risk calculators according to the new European Society of Cardiology guideline on cardiomyopathies [1]

Cardiomyopathy	Risk calculator	Parameters
Hypertrophic cardiomyopathy (HCM)	HCM risk SCD^a^	Age
		Maximum LV wall thickness
		Left atrial size
		LVOT gradient
		Family history of SCD
		NSVT
		Unexplained syncope
Arrhythmogenic right ventricular cardiomyopathy (ARVC)	ARVC risk^b^	Sex
		Syncope
		Number of inverted T‑waves
		Amount of PVC/24 h
		Non-sustained VA
		RVEF
		PES (patients without prior sustained VA)
DCM/laminopathy (LMNA)	LMNA risk VTA calculator^c^	Sex
		Non-missense LMNA mutation
		AV-Block
		Non-sustained VA
		LVEF
DCM/phospholamban (PLN)	PLN risk calculator^d^	LVEF
		Amount of inferior or precordial leads with negative T‑waves
		Low-voltage ECG
		Amount of PVC/24 h

^a^https://qxmd.com/calculate/calculator_303/hcm-risk-scd, as viewed on 27/09/2023

^b^https://arvcrisk.com/, as viewed on 27/09/2023

^c^https://lmna-risk-vta.fr, as viewed on 27/09/2023

^d^https://plnriskcalculator.shinyapps.io/final_shiny/, as viewed on 27/09/2023

## Hypertrophic cardiomyopathy and selected phenocopies

The guideline provides a focused update of the 2014 ESC guidelines on diagnosis and management of HCM [67] considering advances in genetics, cardiac imaging, as well as novel therapies such as cardiac myosin inhibitors. The annual incidence for cardiovascular death in HCM is around 1–2%, with SCD, heart failure, and thromboembolic events in the presence of a high AF prevalence (up to 40%) being the main causes of death. Spontaneous ventricular fibrillation (VF) is often associated with fatal events; however, asystole, AV block, and pulseless electrical activity have also been documented. Of note, most HCM patients are asymptomatic and have a normal lifespan [1] but should be monitored on a regular basis (including ECG and Holter monitoring). A concise assessment of SCD risk is a key part of clinical management and a detailed discussion of clinical features associated with increased risk of SCD is provided (age, non-sustained VT, maximum LV wall thickness, family history, syncope, left atrial diameter, and LVOT diameter). There is lack of randomized data to support pharmacological therapy for prevention of SCD. One small study [68] suggested a lower incidence of SCD with amiodarone [40, 51] but other observational studies [69] found no such effect. Nevertheless, beta-blockers and/or amiodarone are recommended in patients with an ICD and recurrence of symptomatic VA, paroxysmal AF, or repeated shocks despite optimal treatment and ICD programming. Regarding risk stratification, the validated HCM risk-SCD calculator (https://qxmd.com/calculate/calculator_303/hcm-risk-scd) is recommended as the primary tool for estimating risk of sudden death at 5 years and guiding ICD implantation in primary prevention (Class I). It is recommended to assess the risk initially, and thereafter at 1‑ to 2‑year intervals or when a change in clinical status occurs (Class I). Based on risk score and further clinical risk factors (LVEF, LGE), implantation of an ICD may be considered with shared decision-making (Class IIa for high risk > 6%, Class IIb for intermediate risk between 4% and 6%). For the low-risk category (< 4%), the presence of extensive LGE (> 15%) may be considered in shared decision-making (Class IIb). For secondary prevention of SCD, ICD implantation is recommended after cardiac arrest due to VT/VF, or after sustained VA causing syncope or hemodynamic compromise. The role of subcutaneous ICD (S-ICD) in HCM with a potentially increased rate of inappropriate shocks is a matter of debate [70, 71].

**Fig. 4:**
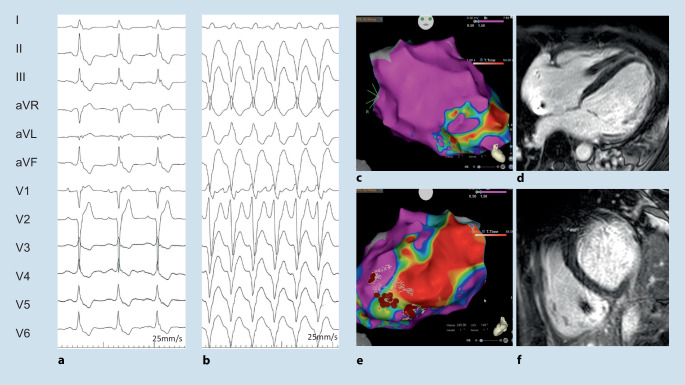
A 51-year-old male with genetically proven Anderson–Fabry disease and a history of aborted sudden cardiac death 4 years before presentation. After initial diagnosis of an assumed viral myocarditis, the patient presented 4 years later to the authors’ department with recurrent ventricular tachycardia (VT) (**b**). Late-stage Fabry disease with cardiac, neurological, and renal impairment was diagnosed. Cardiac magnetic resonance imaging (**d**,**f**) demonstrated apical and extensive posterolateral scarring that correlated to electroanatomical bipolar CARTO maps (**c**,**e**). The patient underwent endo- and epicardial VT ablation

*Anderson–Fabry disease* with inborn deficient or absent enzyme alpha-galactosidase A may be a clinically challenging phenocopy of HCM (Fig. 1). It is often diagnosed in patients with left ventricular hypertrophy and additional cardiac and extracardiac red flags [1]. Bradycardia, chronotropic incompetence, short PQ intervals, and first to third degree AV blocks have been described. AF as well as VA may occur in patients with late diagnosis (Fig. 4).

**Fig. 5:**
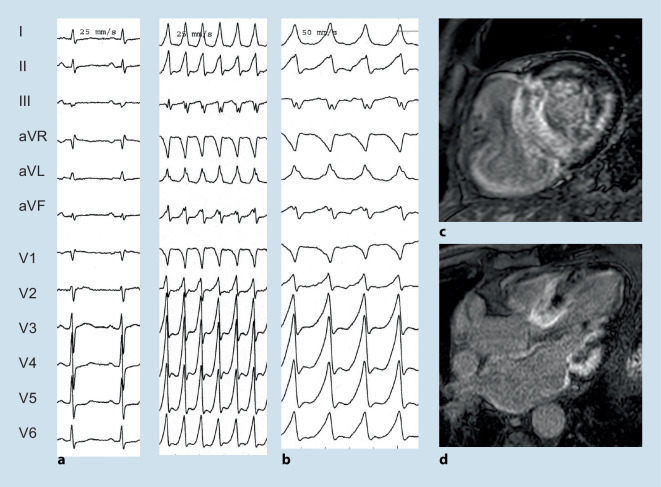
Electrocardiogram (**a**) of a 74-year-old male with wild-type ATTR amyloidosis initially presenting with a sustained ventricular tachycardia (**b**). Note the prominent diffuse late gadolinium enhancement on cardiac magnetic resonance imaging (**c**,**d**)

*Cardiac amyloidosis* is another potential phenocopy of HCM in the form of an RCM that deserves special consideration in cardiac electrophysiology (Fig. 5). Electrical conduction disease with symptomatic bradycardia and advanced AV block or pseudo Q waves may be red flags for amyloidosis. Data on specific antiarrhythmic therapy including ablation for AF or VT and/or ICD implantation for primary prevention is too limited for evidence-based recommendations. The role of non-specific antiarrhythmic therapy such as transthyretin stabilization for AF and/or VT/VF prevention will hopefully be clarified in the next years.

## Arrhythmogenic right ventricular cardiomyopathy (ARVC)

The term ARVC—originating from the pre-genetic and pre-CMR era—already indicates the interaction of arrhythmias with a cardiomyopathy. ARVC usually manifests at the second to fourth decade of life and is characterized by fibro-fatty replacement of myocardial fibers resulting in a fertile environment for arrhythmias. Though many patients with ARVC have left ventricular involvement or may even present with only LV disease (left dominant ARVC) the new guideline has preserved the term arrhythmogenic “right ventricular” CM. This may be debatable in view of concealed and subclinical as well as left-dominant disease forms. Some physicians nowadays prefer the more umbrella like term “arrhythmogenic CM.” However, the task force does not recommend its use as it lacks a morphological or functional definition consistent with the guidelines proposed classification scheme. Moreover, a significant number of conditions, which could be defined as ACM, are now classified as NDLVC.

**Fig. 6:**
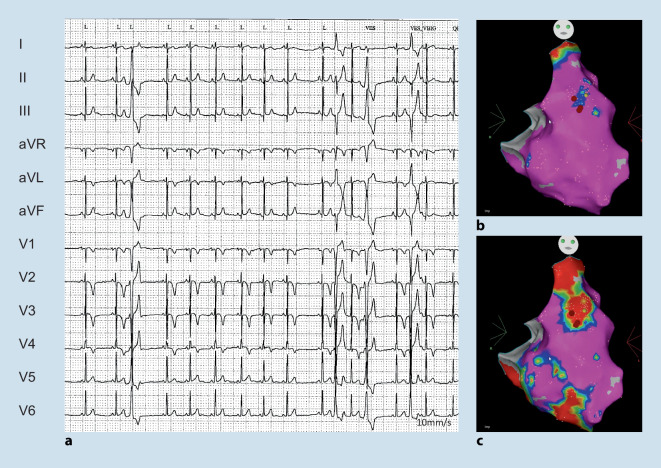
A 21-year-old female competitive athlete who presented with a syncope while playing handball. The electrocardiogram (**a**) was already suspicious for arrhythmogenic right ventricular cardiomyopathy (right precordial negative T waves and VEs with superior and inferior axis). Please also note the unspecific signs of inferolateral early repolarisation. During programmed electrical stimulation, a sustained ventricular tachycardia (VT) with left bundle branch block inferior axis was inducible. Electroanatomic bipolar (color range 0.5–1.5 mV) (**b**) and unipolar (color range 5–8 mV) maps illustrate the predominately epicardial low voltage areas. The VT was successfully ablated from the endocardial anterior right ventricular outflow tract (**c**)

The overall prevalence of ARVC is around 0.08%. Many patients initially present with ECG abnormalities and VA. Of note, cardiac sarcoidosis may be an important phenocopy ([18–20]; Fig. 3). In addition, in up to 20% of patients with acute myocarditis ARVC is diagnosed [1]. Characteristic ECG findings include T wave inversion in V1–V3 with terminal activation delay often combined with atypical right bundle branch block ([19, 72]; Fig. 6). Ventricular extrasystoles (VEs) and/or VT with left bundle branch block (LBBB) morphology (with a superior axis) are a hallmark and must be distinguished from idiopathic RVOT VEs/VT with LBBB and inferior axis [5, 73]. AF is also relatively common with a prevalence around 9–30%. Rhythm control is preferred in the case of symptoms and/or heart failure/LV dysfunction [1].

**Fig. 7:**
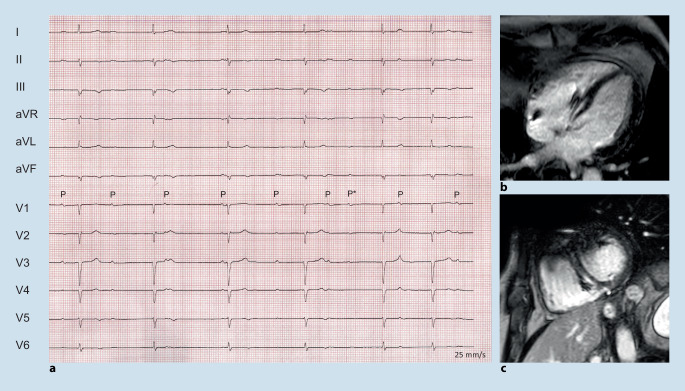
Complete atrioventricular block (**a**) in a 40-year-old male with non-ischemic cardiomyopathy with mildly reduced left ventricular function but prominent intraseptal late gadolinium enhancement (**b**,**c**); the patient was found to have a likely pathogenic laminopathy (Lamin A/C) gene mutation. Marked *P*: sinus p waves and one P* atrial extrasystole in the ECG illustrate complete AV-dissociation in (**a**)

Data on antiarrhythmic therapy in asymptomatic ARVC is scarce but there is good evidence for beta-blocker therapy in symptomatic patients with VEs or VT (Class I). If beta-blockers fail to control arrhythmia-related symptoms flecainide or amiodarone are options (Class IIa). Patients with a sustained VT—even if hemodynamically tolerated—are candidates for an ICD (Class IIa). There is some evidence that ARVC patients may also benefit from S‑ICD [74], but due to lack of pacing most physicians prefer conventional ICDs. In general, pregnancy in ARVC has not been associated with negative-long term outcome, but previous VT in pregnant women represent a WHO risk class III indicating close follow-up at an expert center. Patients with high-risk features (i.e., arrhythmic syncope, non-sustained VT, RVEF < 40%, LVEF < 45%, sustained VT with programmed electrical stimulation [PES]) should also be offered an ICD (Class IIa) (see also arvcrisk.com; Table 2). Of note, the role of PES is not well defined in those who are asymptomatic illustrating some parallels to other fields in electrophysiology such as primary electrical diseases (e.g., Brugada syndrome [75–77]). In the case of incessant VT or frequent ICD interventions catheter ablation (with availability of epicardial approach) should be considered (Class IIa). In cardiac sarcoidosis, VT ablation may also reduce episodes and shocks, but the VT recurrence rate is relatively high [78, 79]. Based on disease acceleration with high-intensity exercise, the ESC guideline recommends against competitive sports in ARVC (Class III) including those who are genotype positive/phenotype negative (with less evidence: Class IIb).

## Dilated cardiomyopathy and non-dilated left ventricular cardiomyopathy

The etiology of DCM is heterogenous and includes inherited and acquired forms. The guideline focuses mainly on genetic DCM. The diagnostic work-up of these patients and especially the results of CMR and genetic testing are relevant for risk stratification and management of VA. A recent retrospective study demonstrated a higher rate of major antiarrhythmic events in patients with genetic DCM variants compared to genotype-negative patients [80]. In certain genotypes, an increased arrhythmogenic risk was observed irrespective of LVEF [80]. The following genes are, for example, associated with increased risk: LMNA, PLN, FLNC, RBM20, EMD, TMEM43, DSP, DSG2, DSC2, and PKP2 [80–84]. In addition to these genetic variants, myocardial scaring determined by LGE on CMR has been established as a strong risk marker for all-cause mortality and VA, both in prospective and retrospective studies. Certain LGE distribution patterns, for example, a “ring-like” pattern, have also been found to be highly prognostic for VA [7, 85].

Regarding primary prevention of SCD the Task Force points out conflicting data from randomized trials, which have included patients with a LVEF < 35%, but overall, an only modest survival benefit in patients with ICD in DCM is observed, as demonstrated by a recent meta-analysis [86]. Nevertheless, patients with high-risk genotypes and additional risk factors (syncope, LGE) should be considered for ICD implantation, even if no LV dysfunction is evident (Class IIa). If no risk factors are present an ICD may even be considered in genotype-positive patients with a LVEF is > 35% (Class IIb). In contrast, in genotype-negative patients with a LVEF > 35%, an ICD may be only considered if risk factors (syncope, LGE) are present (Class IIb). In patients with LVEF ≤ 35% and symptomatic heart failure after > 3 months of optimal medical therapy, an ICD should be considered (Class IIa).

The management of patients with NDLVC regarding prevention of SCD is identical to patients with DCM, albeit with a lower evidence level, mirroring the lack of randomized trials in patients with absent or up to moderate LV dysfunction. Of note, the significance of resting and ambulatory ECG is highlighted for patients with NDLVC, as specific ECG features may suggest an underlying genetic cause (Fig. 1). Therefore, ambulatory ECG monitoring is recommended in patients with NDLVC annually or when there a change in clinical status (Class I). For specific genotypes, namely LMNA and PLN, validated risk calculators are recommended (Table 2). In patients with unexplained syncope, PES may provide useful information concerning the underlying cause: in view of lack of very conclusive data supporting the use of PES for risk stratification it experiences a renaissance [2–4]. In summary, the guideline leaves a lot of room for individualized and shared decision-making regarding ICD implantation, but there is no Class I recommendation for primary prevention.

## Conclusions

Next to other areas of specialization, cardiac electrophysiology plays an integral role in the management of patients with CM. A multidisciplinary and expert approach to CM “has the patient and its family at its heart” [1]. Important innovations of the new guideline include a new phenotype-based classification system with emphasis on a multimodal imaging approach for diagnosis and risk stratification. Patient pathways guide clinicians from the initial presentation to diagnosis. Whilst providing general guidelines for CM management, the primary objective of the new guidelines is to ascertain the disease etiology for facilitating a disease-specific, individualized management.

## Declarations

For this article no studies with human participants or animals were performed by any of the authors. All studies mentioned were in accordance with the ethical standards indicated in each case.
